# Impact of region-of-interest method on quantitative analysis of DTI data in the optic tracts

**DOI:** 10.1186/s12880-016-0145-9

**Published:** 2016-07-11

**Authors:** Ylva Lilja, Oscar Gustafsson, Maria Ljungberg, Daniel Nilsson, Göran Starck

**Affiliations:** Department of Clinical Neuroscience and Rehabilitation, Institute of Neuroscience and Physiology, Sahlgrenska Academy, University of Gothenburg, Gothenburg, Sweden; Ear, Nose and Throat Clinic, Sahlgrenska University Hospital, Gröna stråket 5, 413 45 Göteborg, Sweden; Department of Radiation Physics, Institute of Clinical Sciences, Sahlgrenska Academy, University of Gothenburg, Gothenburg, Sweden; Department of Medical Physics and Biomedical Engineering, Sahlgrenska University Hospital, Gothenburg, Sweden; Department of Neurosurgery, Sahlgrenska University Hospital, Gothenburg, Sweden

## Abstract

**Background:**

To extract DTI parameters from a specific structure, a region of interest (ROI) must be defined. ROI selection in small structures is challenging; the final measurement results could be affected due to the significant impact of small geometrical errors. In this study the optic tracts were analyzed with the aim to assess differences in DTI parameters due to ROI method and to identify the most reliable method.

**Methods:**

Images of 20 healthy subjects were acquired. Fractional anisotropy (FA) was extracted from the optic tracts by four different ROI methods. Manual tracing was performed in 1) the b0 image and 2) a T1-weighted image registered to the FA image. Semi-automatic segmentation was performed based on 3) tractography and 4) the FA-skeleton algorithm in the tract-based spatial statistics (TBSS) framework. Results were analyzed with regard to ROI method as well as to inter-scan, intra-rater and inter-rater reliability.

**Results:**

The resulting FA values divided the ROI methods into two groups that differed significantly: 1) the FA-skeleton and the b0 methods showed higher FA values compared to 2) the tractography and the T1-weighted methods. The intra- and inter-rater variabilities were similar for all methods, except for the tractography method where the inter-rater variability was higher. The FA-skeleton method had a better reproducibility than the other methods.

**Conclusion:**

Choice of ROI method was found to be highly influential on FA values when the optic tracts were analyzed. The FA-skeleton method performed the best, yielding low variability and high repeatability.

## Background

Diffusion tensor imaging (DTI) is a non-invasive MRI technique that can be applied in vivo to detect white matter pathology in nerves and neural pathways of the brain [1]. Prior studies have demonstrated the ability of DTI to detect pathological changes in the anterior visual pathways, for example in patients with pituitary tumors [2] and optic neuritis [3, 4], reflecting underlying microstructural changes.

However, extraction of DTI parameters from small structures, such as the anterior visual pathways, is a challenge. The small size results in an increased difficulty of identifying the correct voxels as well as a risk of including voxels affected by partial volume effect. Previous studies have reported a higher variability of DTI parameters extracted from small structures, compared to that of larger structures [5–10]. To this date, there is no consensus about data extraction method.

**Fig. 1:**
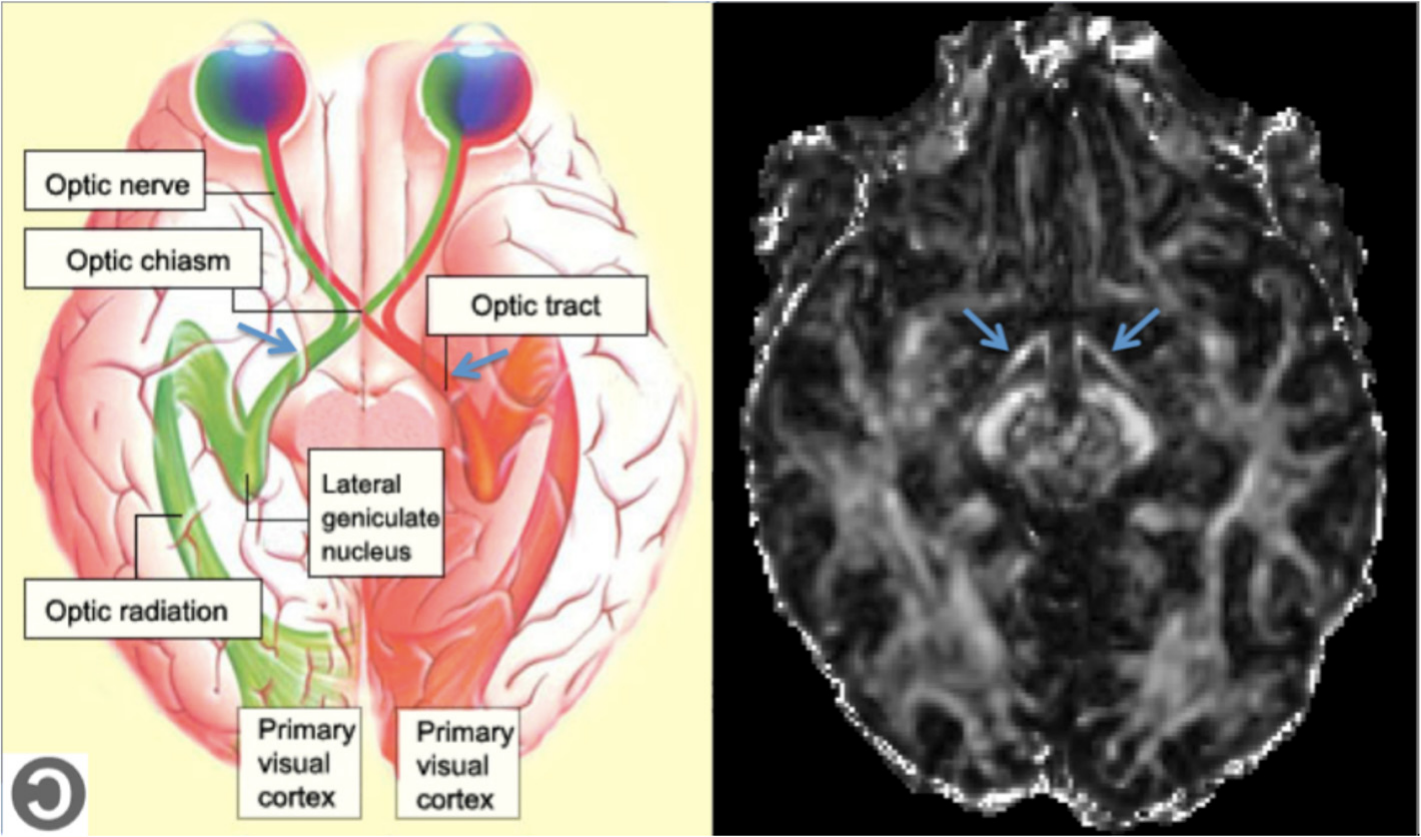
*Left*: schematic illustration of the visual pathways in the brain (Copyleft from http://thebrain.mcgill.ca). *Right*: Axial slice of an FA-map, at the level of the optic tracts. The optic tracts are indicated with *blue arrows* in each image

In this study we focus on the extraction of DTI parameters from the optic tracts (OT) that are part of the anterior visual pathways (Fig. 1). The OTs are an example of small but well-defined structures that are visible in a regular clinical whole-brain DTI scan. Furthermore, the OTs are of specific interest in several pathological conditions affecting the visual pathways and the eyes.

To this date, different methods have been used for the extraction and analysis of local DTI parameters. The most common methods in the literature so far are region-of-interest (ROI) methods, by manual voxel selection based on preexisting anatomical knowledge or voxel selection by tractography. DTI parameters can also be extracted and assessed by group-comparison methods, such as voxel-based morphometry (VBM) [11, 12] and tract-based spatial statistics (TBSS) [13].

Group-comparison methods, such as VBM and TBSS, include registration of all subjects’ scans to a common space. Such registration may perform poorly for structures with certain anatomical properties, for example structures that vary anatomically between subjects, that are relatively small, and that are localized in areas prone to image artifacts. Carefully hand-drawn ROIs (manual ROIs) in original diffusion space have the advantage of adapting to changes between scans, but potentially suffer from subjectivity/user-error. Smith et al. compared inter-scan and inter-subject variability between TBSS, VBM and manual ROIs [13]. TBSS resulted in the lowest variance for most structures while manual ROIs had the lowest variance for some structures. However, the study by Smith et al. focused on white matter tracts that are relatively large, compared to the OTs – the focus of the present study.

In this study, fractional anisotropy (FA) was extracted from the optic tracts by four different ROI methods: two with manual voxel selection and two with semi-automatic voxel selection. The aims were to 1) assess differences in FA and reliability between the methods, and 2) to identify the most stable method for FA extraction in the optic tracts.

## Methods

### Subjects

Twenty healthy subjects with normal vision (apart from refractive errors) were included (age range 30–61 years, mean 44; 7 male). All subjects but two underwent a standardized neuro-ophthalmological examination that confirmed normal vision. The remaining two subjects reported normal vision. One of the subjects was scanned six times on the same day, with repositioning in the scanner preceding each scan.

The study was approved by the regional ethical board of the University of Gothenburg. Informed written consent was obtained from all subjects prior to inclusion in the study.

### Image acquisition

MR imaging was performed on a Philips Achieva 1.5 T scanner equipped with software release 3.2 (Philips, Eindhoven, the Netherlands). An eight-element SENSE head coil was used (same vendor). To reduce head motion, the subjects’ heads were firmly supported with cushions.

A T1-weighted 3D image of the brain was acquired using a TFE scan with isotropic voxel size of 1 mm^3^. SENSE factor was 1.4 in the anterior-posterior (AP) direction and 2 in the right-left (RL) direction. TE/TR/TI = 3.3/7.2/694 ms. NSA = 1. Flip angle = 8°. No fat suppression was used. The scan time was 7 min.

DTI of the whole brain was performed with the following parameters: single shot spin echo echo planar imaging with TE = 69 ms, axial slices, SENSE factor 3.2, half scan factor 0.712, isotropic 2.2 mm × 2.2 mm × 2.2 mm voxels, *b* = 0 s/mm^2^ (6 signals averaged) plus 32 diffusion-sensitizing gradient directions (*b* = 800 s/mm^2^, 3 signals averaged) and phase encoding in the anterior-posterior direction (bandwidth 17.2 Hz/mm). Reconstructed in plane resolution by zero-filling was 1.9 mm × 1.9 mm. The scan time was 16 min.

All images were angulated and positioned to be parallel to the lower limit of the corpus callosum, in the sagittal view, and to the plane of symmetry of the brain in the transversal view.

### Data analysis

FMRIB’s Software Library (FSL) was used for the data post processing (http://fsl.fmrib.ox.ac.uk/fsl/fslwiki). The voxels of the diffusion images were interpolated to an isotropic size of 1 mm^3^ using a sinc-like spline interpolation (FLIRT) [14]. Eddy current and motion correction was then carried out by affine registration to the b = 0 image [14]. The diffusion tensor was calculated in each voxel within the brain using the diffusion toolbox in FSL. To characterize the anisotropy of the diffusion in each voxel, FA was calculated based on the eigenvalues of the diffusion tensor.

### ROI definition – four methods

**Fig. 2:**
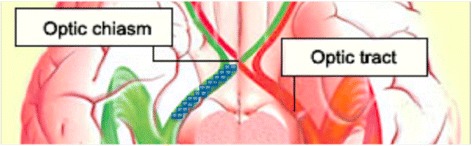
Schematic illustration of an ROI (*blue squares*) of the left optic tract. All ROIs aimed to start at the posterior part of the optic chiasm and to include the central portion of the optic tract. The most anterior 15 mm of all optic tracts could be clearly identified in all subjects, thus the length of an ROI was set to cover a 15 mm anterior-to-posterior distance

All four ROI methods were designed to define voxels corresponding to the central portions of the OTs and to avoid inclusion of border zone voxels that could be influenced by partial volume effect. Thus, a maximum of two voxels were selected per coronal slice (i.e., cross section of the OT) for each ROI. The anterior limit of all ROIs was defined as the most posterior part of the optic chiasm, which was identified by visual inspection of coronal slices of color-coded FA maps. Within individual, the same coronal slice was used as the anterior limit for all four ROI methods. For the two manual methods, voxel selection proceeded posteriorly, slice by slice, continuously, until visual inspection no longer could separate the OTs from other structures reliably. Figure 2 illustrates a typical ideal ROI.

ROIs of the right and the left OT were defined separately. Two clinicians (YL and DN) with previous DTI experience defined all ROIs separately, as well as the masks for tractography, for inter-rater assessment purpose. The procedure was repeated once by one of the clinicians (YL) for intra-rater assessment (procedure time points separated by two months).

#### Manual tracing in b = 0 image (“Manual b0”)

**Fig. 3:**
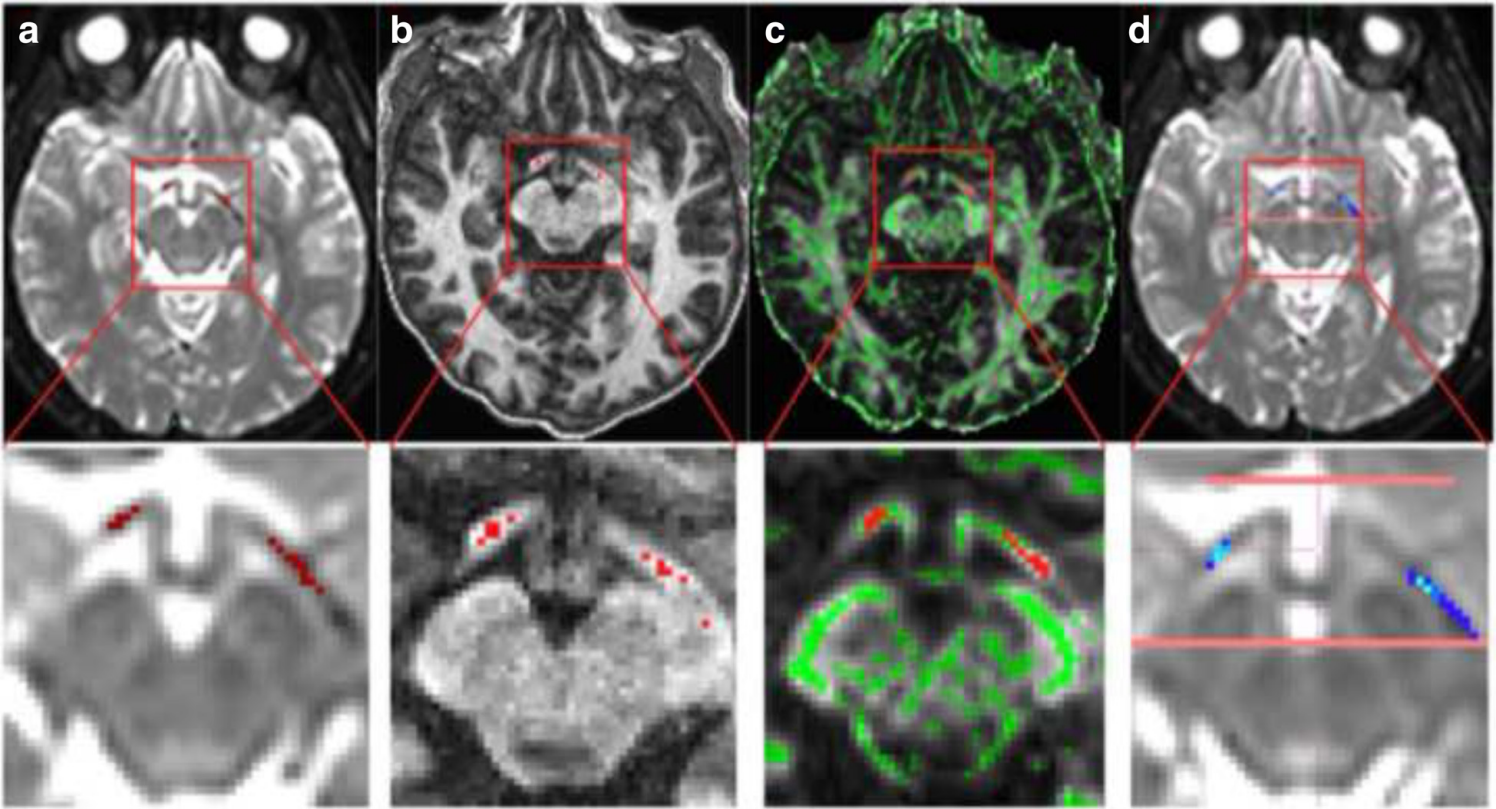
Illustration of the four ROI methods (*upper row*: axial slices of whole MRI images; *lower row*: close-ups of the optic tracts and ROIs). From the left: **a** manual ROI in b0 image, **b** manual ROI in coregistered T1-weighted image, **c** ROI guided by FA-skeleton (FA-skeleton in green) and **d** ROI guided by probabilistic tractography. The pink lines in the tractography image represent the termination masks of the tractography. A complete ROI cannot be shown in a single axial slice as the optic tracts are oriented anatomically in an inferior-superior direction, thus only parts of the ROIs are seen in this image (indicated as *red* or *blue* voxels)

ROIs were defined manually by visual inspection of the b = 0 map. The two most central voxels of the OT, in each coronal slice, were selected (Fig. 3).

#### Manual tracing in coregistered T1-weighted image (“Manual T1W”)

For each subject the T1-weighted image was registered to the subject’s FA map using a free form deformation method [15]. Warping the T1-weighted image to diffusion space, and not the other way around, was chosen in order to avoid additional degrading effects from interpolation in the diffusion-weighted images of the FA map. Normalized mutual information was used as similarity measure. ROIs were defined by manual tracing in the coregistered T1-weighted images. The two most central voxels of the OT, in each coronal slice, were selected (Fig. 3).

#### Semi-automated method based on FA skeleton (“FA-skeleton”)

The skeleton algorithm in TBSS was applied on each FA map, keeping the original space of each individual FA map [13]. Voxels with FA < 0.2 were excluded from the resulting FA skeleton in order to exclude voxels that were primarily gray matter or CSF. Voxel selection was restricted to voxels included in the FA skeleton that represented the OT. A maximum of two voxels of the FA skeleton per coronal slice were selected. When the OT was represented by more than two voxels per coronal slice, two voxels with high FA representing the middle of the tract were selected by visual inspection. When the OT was represented by a single voxel, only this voxel was chosen (Fig. 3).

#### Semi-automated method based on probabilistic tractography (“Tractography”)

Probabilistic tractography was carried out by the BEDPOSTX and the PROBTRACKX tools in FSL (version 5.0.4) [16], using default settings. Seed masks were defined as single voxels in the OTs, approximately 5 mm posterior of the optic chiasm. Termination masks were defined in the posterior section of the optic chiasm (using the same coronal slice selected as the start slice for the other ROI methods) and as a large coronal mask including the lateral geniculate nuclei. An exclusion mask was defined in the cerebral peduncles, in the axial plane at a level just inferior to the OTs. In each coronal slice of the resulting tractographies, the two voxels with the highest FA values were selected to constitute the ROI (Fig. 3).

### Statistical analysis

#### Comparison of ROI methods

In all images it was possible to identify the OTs to a point at least 15 coronal slices posterior of the optic chiasm, thus these 15 slices were selected for statistical analysis. In order to compare measurements of FA between ROIs the OTs were divided into lengthwise sections of 5 mm (anterior-posterior direction), starting from the most anterior voxels of each OT ROI.

The FA was averaged within these sections and across sides for each subject. The assumption of normality was tested and confirmed by visual inspection of Q-Q (Quantile-Quantile) plots. The mean, standard deviation (SD) and coefficient of variation (CV, SD/mean) over all subjects were calculated for each section.

To test for differences in measured FA between ROI methods a covariance pattern model was set up, which included the effects of multiple measurements from the same subject given by the five slices within a section. Pairwise comparisons between ROI methods were conducted for each section. This gave a test similar to the paired t-test with unequal variances. The results were adjusted for multiple comparisons using the Tukey-Cramer method.

#### Intra- and inter-rater reliability

Repeatability coefficients and limits of agreement were calculated for each coronal slice of the ROIs in order to quantify the intra- and inter-rater reliability respectively [17]. A two-way ANOVA, considering subject and rater as random effects and side as a fixed effect, was used to estimate the variance components due to the different effects and their interactions. This accounted for the multiple measures within each subject (right and left OT). The repeatability coefficient was calculated as $1.96\kern0.1em \cdotp \kern.7em \sqrt{2}\kern0.1em \cdotp \kern.8em {\sigma}_{error}$, where *σ*_*error*_ is the error term in the ANOVA [17]. The limits of agreement were calculated as $\overline{d}\pm 1.96\kern0.2em \cdotp \kern0.8em \sqrt{2}\kern0.2em \cdotp \kern0.8em {\sigma}_{sum, rater}$, where $\overline{d}$ is the mean difference and *σ*_sum,rater_ is the square root of the sum of all variance components for interaction terms including rater, together with the error term [17].

In order to explore the constituents of variability, two additional tests were performed: 1) correction for differing starting points between ratings (next) and 2) Jaccard analysis section.

Intra- and inter-rater analyses were also performed after correction of starting point, i.e., by finding the most anterior slice included in both of the compared ROIs and setting this slice as the start point. The purpose of this analysis was to remove the effect of varying start slices, in order to investigate the variability within each specific coronal slice and to assess variability due to differing starting points. This correction could only be accomplished for repeated analysis of the same data set and was thus impossible for comparisons between subjects or repeated scans.

#### Inter-scan variability

The inter-scan variability was calculated in a similar way as the inter-rater reliability. Side was considered a fixed effect whereas scan number was considered a random effect. The variance term due to scan number was summed with the error term to form the total variance due to scan number.

#### Jaccard analysis

With the aim to further investigate constituents of differences between ROI methods, Jaccard index was used. This index is defined as the number of voxels where two ROIs overlap (i.e., where the same voxels have been selected), divided by the number of voxels that any of the two ROIs have included. As a result, the Jaccard index ranges from zero, which is no overlap at all, to one, which is complete agreement. In order to reduce the effect of different positions of first and last slice, the Jaccard index was only calculated over slices included in both ROIs. Note that complete agreement cannot be accomplished if the ROIs are of different sizes.

## Results

### Comparison of ROI methods

**Fig. 4:**
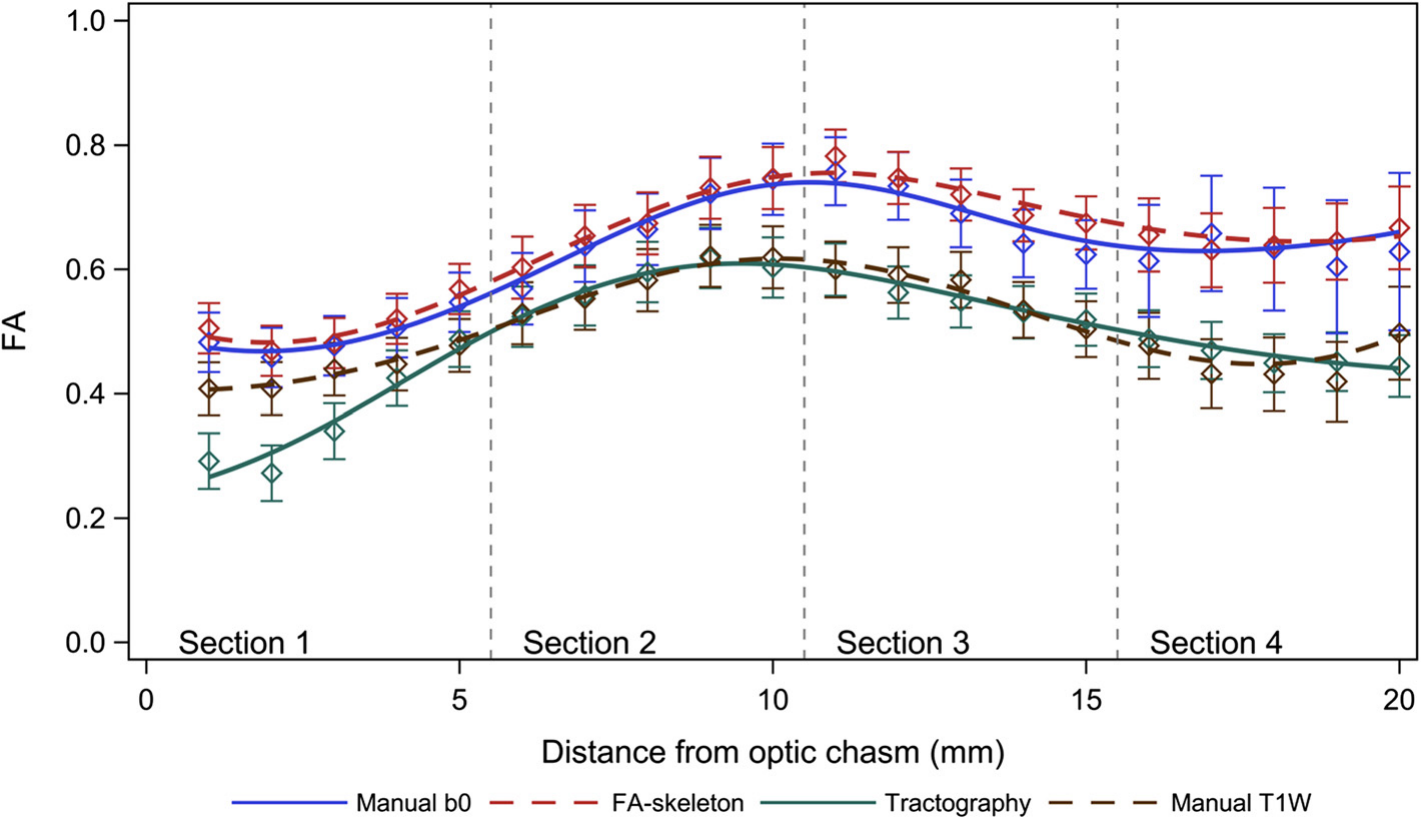
Mean and 95 % confidence interval (CI) of FA values per position, for all four ROI methods separately. Overall trends are represented as splines fitted to data

There were no significant differences of FA between the sides (left and right OTs), therefore the averaged FA over both sides was used for further comparisons between methods. The resulting FA values divided the ROI methods into two groups that were significantly different from each other (corrected *p* < 0.05), but within each group there were no significant differences: 1) the manual b0 and the FA-skeleton methods and 2) the manual T1W and the tractography methods (Fig. 4, Table 1). The latter was true for all sections but Background section (the most anterior 5 mm) where the tractography and the manual T1W methods differed significantly. FA values were higher using the FA-skeleton and the manual b0 methods compared to the tractography and the manual T1W method, for all OT sections (Table 2).

**Table 1:** Pairwise comparison of ROI methods, by mean FA across both sides, per section of the optic tracts

Method 1	Method 2	Section 1		Section 2		Section 3	
		Mean (95 % CI)	*p-*value	Mean (95 % CI)	*p-*value	Mean (95 % CI)	*p-*value
FA-skeleton	Manual T1W	0.07 (0.03; 0.11)	<.0001	0.10 (0.06; 0.14)	<.0001	0.16 (0.11; 0.21)	<.0001
	Manual b0	0.01 (−0.00; 0.03)	0.1501	0.01 (−0.01; 0.04)	0.4104	0.03 (−0.00; 0.07)	0.0559
	Tractography	0.15 (0.10; 0.19)	<.0001	0.10 (0.04; 0.16)	0.0003	0.17 (0.12; 0.22)	<.0001
Manual T1W	Manual b0	−0.06 (−0.10; −0.01)	0.0047	−0.09 (−0.13; −0.04)	0.0001	−0.13 (−0.18; −0.07)	<.0001
	Tractography	0.07 (0.02; 0.13)	0.0051	0.00 (−0.06; 0.06)	0.9999	0.01 (−0.04; 0.06)	0.9573
Manual b0	Tractography	0.13 (0.08; 0.18)	<.0001	0.09 (0.02; 0.15)	0.0046	0.14 (0.08; 0.20)	<.0001

Pairwise difference in FA between ROI methods. Confidence intervals and *p*-values were corrected for multiplicity using the Tukey-Cramer method

**Table 2:** Mean, standard deviation (sd) and coefficient of variance (CV) of FA by the four ROI methods separately

ROI method	Section 1		Section 2		Section 3	
	Mean ± sd	CV %	Mean ± sd	CV %	Mean ± sd	CV %
Manual b0	0.50 ± 0.09	18	0.67 ± 0.11	17	0.70 ± 0.09	12
FA-skeleton	0.51 ± 0.08	16	0.68 ± 0.09	14	0.73 ± 0.06	8
Manual T1W	0.44 ± 0.08	18	0.58 ± 0.09	16	0.56 ± 0.06	11
Tractography	0.41 ± 0.10	24	0.63 ± 0.11	18	0.62 ± 0.10	16

### Intra- and inter-rater reliability

The mean difference between measurements from the two raters (inter-rater) was found to be close to zero for all methods and positions.

**Fig. 5:**
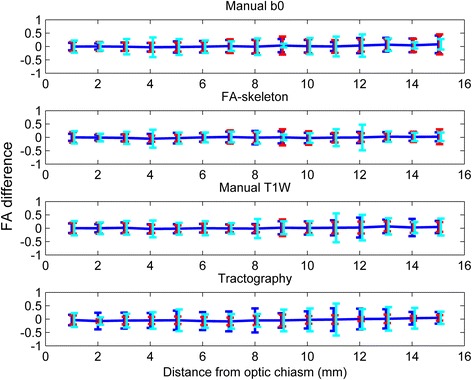
Inter-rater (*blue*), intra-rater (*red*) and scan variability (*cyan*) for all methods and position. The repeatability coefficient (intra-rater) and scan variability were expressed as an interval with the same midpoint as the corresponding limit of agreement (inter-rater) to facilitate comparisons

The intra- and inter-rater variabilities were similar within all methods, except for the tractography method where the inter-rater variability was higher. The rater variability of the FA-skeleton method was slightly lower than those of the other methods (Fig. 5).

**Fig. 6:**
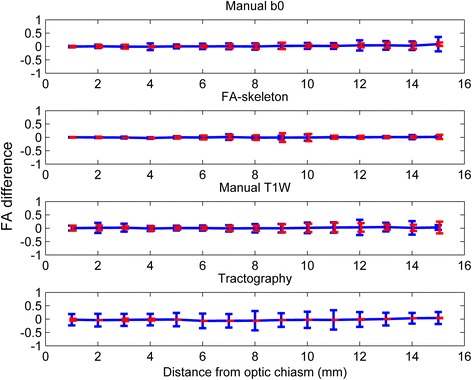
Correction for position by redefining the first position in each ROI as the most anterior coronal slice included in both ROIs. Inter-rater (*blue*) and intra-rater variability (*red*) for all methods and position. The repeatability coefficient (intra-rater) was expressed as an interval with the same midpoint as the corresponding limit of agreement (inter-rater) for easier comparisons

Almost all the variability between measurements within and between raters was explained by different starting points, which can be seen in Fig. 6, where varying starting point was corrected for. This effect was especially pronounced for the manual b0 and the FA-skeleton methods. For the manual T1W method, varying start points explained less of the variability. For the tractography method the repeatability coefficient was reduced to almost zero when a start point correction was applied, whereas the limits of agreement (inter-rater variability) remained almost unchanged.

### Inter-scan repeatability

The inter-scan variability was found to be slightly higher than the inter- and intra-rater variabilities for all methods (Fig. 5).

### Jaccard analysis

When comparing ROI methods the Jaccard indices were in general low (~0.3). The highest Jaccard index between methods was found between the FA-skeleton and the manual b0 method (Table 3).

**Table 3:** Pairwise comparison of ROI methods using Jaccard index

Method 1	Method 2	Jaccard index (mean ± sd)
Manual b0	FA-skeleton	0.42 ± 0.08
	Tractography	0.30 ± 0.11
	Manual T1W	0.27 ± 0.07
FA-skeleton	Tractography	0.31 ± 0.13
	Manual T1W	0.26 ± 0.10
Tractography	Manual T1W	0.28 ± 0.09

## Discussion

### Summary of findings

Choice of ROI method was found to significantly affect the FA values when the optic tracts were analyzed. The manual b0 and the FA-skeleton methods resulted in the highest FA values, indicating that these two methods best identified the middle of the optic tracts, where voxels are less influenced by partial volume effects. Results from the FA-skeleton method had lower variability compared to the manual b0 method for all comparisons (inter-individual, inter-scan and intra- and inter-rater analysis). This suggests that the FA-skeleton method, compared to the manual b0 method, leads to more reliable results.

Interestingly, the manual T1W and the tractography methods resulted in similar FA values, which were significantly lower than for the other two methods, indicating an inability to accurately define the middle of the small structures that are the optic tracts.

A suitable ROI method, for both clinical and research purposes, should have an ability to accurately define the structure of interest and a high reliability. Based on the results of the present study the FA-skeleton ROI method performed best according to these criteria and may be suggested for analysis of the optic tracts, and possibly of similar structures, with regard to size, shape and expected image artifacts.

### Comparison of ROI methods

ROIs for DTI may be defined in images with more anatomical information, such as T1-weighted images, which are subsequently registered to diffusion space; such registration inevitably leads to image distortion to some degree [18, 19]. Grech-Sollars et al. coregistered b0 images to high-resolution T1-weighted images, preceding registration to standard space (MNI) [19]. They found acceptable overall FA values, however, they also found very low values for specific white matter structures, such as the optic chiasm (FA 0.18). These spuriously low values were probably due to the choice of linear registration. In the present study, whole brain coregistration between T1-weighted images and diffusion space, through different attempts of both global and local affine transformations, did not produce a sufficiently good match for the OTs, although other white matter structures were adequately matched. A likely reason for this registration failure is the size of the OTs in combination with susceptibility-artifact effects. The most successful match was achieved when a spline-based transformation was applied for registration of the T1-weighted images to the FA maps. Such a transform is able to mimic geometric effects of susceptibility artifacts. However, the FA values extracted by this method were lower than those of the manual b0 and FA-skeleton methods, suggesting a remaining slight off set after the coregistration.

In previous literature, manual ROIs in diffusion image space, such as the b = 0 map or the FA map, have been preferred for analysis of the anterior visual pathways [5, 20, 21]. Hakulinen et al. compared two different manual ROI methods – circular and free hand – on several white matter structures [8]. They found that FA differed depending on ROI method and, in accordance with other studies, that the variability was larger the smaller the structure [5–7, 9, 10]. This effect for small structures is most likely due to the measurement procedure: the difficulty of redefining similar ROIs, leading to user-errors, and the increased risk of including boarder-zone voxels affected by partial volume effects.

More objective data extraction methods could resolve the issue of user-errors. One such proposed method is data extraction by tractography, where part of the resulting tract can be selected for data extraction. Paul et al. used probabilistic tractography to visualize and extract data from the OTs [2]. They reported differences in DTI parameters between patients with pituitary tumors, affecting the anterior visual pathways and vision to varying degrees. However, tractographies have been shown to differ to a significant degree depending on tractography algorithm as well as on the selected parameters within the algorithm [22]. For example, Wang et al. assessed reliability of DTI parameters by tractography-based ROIs, comparing 15 and 30 gradient sampling directions, and found significantly affected values as well as reduced variability using 30 directions [23].

In the current study, the results by tractography-based ROIs showed a strong intra-rater agreement but a poor inter-rater reproducibility (Fig. 5). The only factors that differed between different raters were the manually chosen seed, termination and exclusion masks; although carefully anatomically defined, the differences that resulted between raters proved to have an important effect on the tractographies. Reproducibility could thus be a problem for tractography-based ROIs in small structures, which should be considered in comparison of results from different studies.

In this study, we propose a version of ROI method that aspires to bypass issues of subjectivity/user-errors, registration and tractography. The skeleton algorithm of TBSS was applied on each subject’s individual FA map, keeping the original image space. This creates an FA skeleton of the entire brain for each individual, defining the central voxels of each white matter tract [13]. The user defined start and end of the OT, but the voxels were otherwise primarily selected by the algorithm. Compared to the other ROI methods in this study, the FA-skeleton method performed well: the mean FA was high, suggesting the middle of the tracts was successfully identified, and the variabilities across subjects and between measurements were low. Since the OTs are thin structures it could be assumed that the voxels with the highest FA include the center of the structures; the FA-skeleton normally defines two voxels per cross section and it is likely that voxels lateral of these most central voxels are affected by partial volume effect, resulting in lower FA. When performing the full TBSS procedure on all subjects, including the registration to a common space, the OTs were not identifiable. The individual FA-skeleton version of ROI method could be an alternative when comparing and extracting data from relatively small structures, and could be used for comparisons where significant anatomical differences are expected.

### Reliability

Rater performance is an important factor of variability. Previous DTI reliability studies have reported significantly higher inter-rater than intra-rater variability [24], similar to results by the tractography method in this study. However, the other three ROI methods herein show little difference between the intra- and inter-rater variability, indicating high reproducibility.

The variability due to repeat-scan was higher than the intra- and inter-rater variabilities, which is to be expected as repeated scans include the intra-rater error as well as scan-specific factors. There are several scan-specific factors that may affect measurements. The most important ones include differing slice and head positioning, where changes between scans will affect the partial volume effects and possibly also the effects of susceptibility. Varying head motion is another possible source of inter-scan variability, especially if the averaging is carried out on scans where intermediate motion may occur, which was the case in the present study [25]. Although the intra-rater variability was lower for the tractography method compared to the other three ROI methods, the inter-scan variability for the tractography method was higher. Possibly, the tractography algorithm is more sensitive to the scan-specific changes.

When adjusting for starting slice, the variability decreased considerably (Fig. 6). The greatest decreases for the combination of intra- and inter-rater variability were seen for the manual b0 and the FA-skeleton ROI methods, which imply that the subjectivity in the identification of the start slice was an important factor of variability, whilst the ROI was otherwise well reproducible. In conclusion, small rater-dependent factors have a large impact on ROI analysis of small structures.

The Jaccard indices were in general low between ROI methods (~0.3), including between the ROI methods that showed high similarity in FA values. At first glance this may seem contradictory, however, Jaccard index only compares chosen voxels, and does not take into account the values within the voxels. The explanation for these findings could be that the ROIs in this study were chosen to be “thin” – only two voxels per coronal OT section. More than two voxels could be representative of the middle of the OT, and thus give similarly high FA values. The choice of two voxels was a compromise between reducing the risk of partial volume effect and increasing the amount of included data*.* In conclusion, the similarities between ROI-method results in this study could be said to be due to their ability, and similarities in ability, to identify the center of the optic tract, but not due to their ability to choose the same voxels.

### Limitations

The anterior limit of the ROIs was defined as the most posterior part of the optic chiasm. Thus, a certain number of chiasm voxels will be included in Background section (i.e., the most anterior section). In the optic chiasm there are several different fiber directions, as fibers corresponding to different parts of the visual fields are reorganized. As a consequence, the FA in such voxels will be an average of several fiber orientations. The diffusion tensor model used herein, which assumes one principal diffusion direction per voxel, may thus be poorly suited for the chiasm, including Background section. However, the tensor model is well suited for the parallel organization of the OTs, and the focus of this study should thus be on results from Methods and Results sections.

Because of magnetic susceptibility effects in the region of interest in this study we used a 1.5 T scanner, instead of a 3 T, with a relatively high SENSE factor (=3.2). Both of these choices contribute to mitigate image distortion caused by magnetic susceptibility, and result also in lower SNR. Therefore increased signal averaging was used to enhance the SNR. More recent techniques for reduction of image distortions, such as zoomed acquisition, were not available at the site by the time of the study.

A single scan protocol and resolution was applied in this study. The relationships between ROI-methods reported herein may be different for other protocols, for example protocols with smaller voxels or a different number of diffusion encoding directions. However, the spatial resolution, field of view, scan time and number of gradient direction selected for this study are commonly used for FA-study and tractography purposes.

## Conclusion

The choice of ROI method was found to be highly influential on the resulting FA values when the optic tracts were analyzed. Both reliability and magnitude of FA values varied due to ROI method. The FA-skeleton method proposed herein proved stable between measurements and able to well locate the structure of interest. The spread in results in this study shows that an awareness of the effect of methodology in the analysis of DTI parameters is essential.

## Abbreviations

DTI, Diffusion tensor imaging; FA, Fractional anisotropy; MNI, Montreal neurological institute; OT, Optic tract; ROI, Region of interest; SNR, Signal to noise ratio; T1W, T1-weighted; TBSS, Tract-based spatial statistics; VBM, Voxel-based morphometry
